# Chicago Medical Society

**Published:** 1879-10

**Authors:** 


### ARTICLE X.

CHICAGO MEDICAL SOCIETY. Regular meeting September 1st, 1879.

DR. R. PARK read a paper on "Dermatitis venenata," describing the poison ivy and giving at length its characteristics, chemical constituents and properties, with a full account of the symptoms caused by it, their duration, termination, pathology, differential diagnosis and treatment. The paper, besides giving his own experience with it, was a résumé of all the literature that he could get access to.*

In the paper the ground was taken that the eruption was simply a specific eczema, *i. e.*, an eczema with a specific ætiology,—the toxicodendric acid,—and had for its commonest type the vesicular form of the same. While there were variations in all directions from this standard, it was nevertheless the prevailing one.

With respect to the erysipelatous nature of the symptoms, as alleged by some writers, the essayest took the ground that it was

* This paper appeared in full in the "Archives of Dermatology," July, 1879.

very rare. Concerning treatment, he mentioned a great variety of drugs, both vegetable and mineral, which had received the recommendation of one or another, giving such an extended list more as a matter of curiosity than of practical utility, however, and advising those who have occasion to treat the malady to select a very few remedies on which they could depend, and use them judiciously. He recommended the camphor-chloral mixture as a powerful sedative to relieve the distressing itching, stating at the same time that it was not in the slightest astringent or antiphlogistic. Hypnotics and anodynes were recommended, and saline laxatives *pro re nata*.

In the discussion which followed, DR. HAMILL stated that he had been called upon to treat many of these cases, and had always used an ordinary brine locally and with great success. This brine might be made with common salt, or saltpetre, or alum might be added.

DR. PAOLI insisted on the erysipelatous nature of many of the cases which he had seen formerly in Ohio, saying that in many of them there was very little of the vesicular appearance, while they did present the peculiar color and œdema of erysipelas. In many of them also large superficial ulcerations accompanied or followed the other symptoms.

DR. T. D. FITCH thought the essayist had not laid sufficient stress upon the use of laxatives; he also saw indications in this grouping of symptoms for arterial sedatives. He narrated the case of a lady suffering from ivy-poisoning who, during its course, had an hæmatocele in the right mamma. He had seen very little of anything like erysipelas in his cases.

DR. WARE stated that in his boyhood it was a very common thing for his schoolmates to be affected, sometimes very severely. No physician was called, however; some simple domestic treatment was adopted, and in a few days they were as lively as usual.

Several others mentioned cases they had seen or remedies they had tried. In conclusion,

DR. PARK said he was not prepared to admit the erysipelatous nature of the affection. Those cases which so closely resembled it he thought should be termed *erysipelatoid*. He could understand how a patient suffering from the effects of poison-ivy could be attacked with a genuine erysipelas; also that constitutional conditions might make no small difference in the characteristics of the disease; but he claimed that the picture he had drawn of the affection was in the main true and correct, and that the limits of his paper did not permit mention of every possible variation. He also held that erysipelas was a disease with a specific ætiology, of which we were yet ignorant, usually manifested at first by a chill and other significant symptoms; that in the dermatitis under discussion peripheral sepsis might cause lymphangitis or perilymphangitis, which could give rise to the appearances with which all were familiar in true erysipelas.

He said, with reference to the use of laxatives, that he thought a simple mention of their advisability would be enough to govern the physician in charge of any case of this kind. Finally, he regarded the high fever and accompanying symptoms as due very largely to reflex disturbence from peripheral irritation which would, and generally do, subside upon exhibition of the proper sedatives, general and local.

The discussion of the paper being concluded, DR. C. T. FENN showed a very interesting specimen of extra-capsular fracture of the neck of the femur removed nineteen months after the injury. The specimen showed grooving from pressure of tendons, some changes in the shape of the parts and an absorption of part of the shaft, allowing the neck of the bone to encroach upon and extend into the medullary cavity. The specimen was accompanied by a full history of the case.

